# Rechargeable Multifunctional Anti‐Bacterial AEMs for Electrodialysis: Improving Anti‐Biological Performance via Synergistic Antibacterial Mechanism

**DOI:** 10.1002/advs.202303588

**Published:** 2023-09-11

**Authors:** Yuyang Yao, Junjie Mu, Yuan Li, Yanjing Ma, Jingwen Xu, Yuna Shi, Junbin Liao, Zhenlu Shen, Jiangnan Shen

**Affiliations:** ^1^ College of Chemical Engineering Zhejiang University of Technology Hangzhou 310014 China; ^2^ Information Materials and Intelligent Sensing Laboratory of Anhui Province Institutes of Physical Science and Information Technology Anhui University Hefei 230601 China; ^3^ College of Biotechnology and Bioengineering Zhejiang University of Technology Hangzhou 310014 China

**Keywords:** anion exchange membrane, anti‐biofilm, electrodialysis, surface modification, synergistic antibacterial mechanism

## Abstract

Constructing a functional layer on the surface of commercial membrane (as a substrate) to inhibit the formation of biofilms is an efficient strategy to prepare an antibacterial anion exchange membrane (AEM). Herein, a rechargeable multifunctional anti‐biological system is reported by utilizing the mussel‐inspired L‐dopa connection function on commercial AEMs. Cobalt nanoparticles (Co NPs) and N‐chloramine compounds are deposited on the AEM surface by a two‐step modification procedure. The anti‐biofouling abilities of the membranes are qualitatively and quantitatively analyzed by adopting common Gram‐negative (*E. coli*) and Gram‐positive (*S. aureus & Bacillus*) bacteria as model biofouling organisms. The optimized membrane exhibits a high stability concerning the NaCl solution separation performance within 240 min. Meantime, the mechanism of the anti‐adhesion is un‐veiled at an atomic level and molecular dynamics (MD) simulation are conducted to measure the interaction, adsorption energy and average loading by using lipopolysaccharide (LPS) of *E. coli*. In view of the superior performance of antibacterial surfaces, it is believed that this work could provide a valuable guideline for the design of membrane materials with resistance to biological contamination.

## Graphical abstract

A rechargeable multifunctional anti‐biological fouling anion exchange membrane (AEM) shows effective anti‐biofilm formation and anti‐adhesion performance. The mechanism of the anti‐adhesion are un‐veiled at an atomic level. It is believed that this work can provide a valuable guideline for the design of membrane materials with resistance to biological contamination.

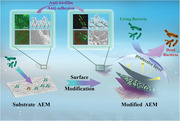

## 1 Introduction

In recent years, sustainable development has become a priority, which requires the exploration of resource recovery and wastewater treatment technologies.^[^ ^1^ ^]^ Membrane separation technology is characterized by high efficiency, selectivity, energy saving, simple operation and environmental friendliness, and has been broadly used in separation, purification, and concentration of biological macro‐molecules such as proteins, polysaccharides, etc.^[^ ^2^, ^3^ ^]^ As the core component of electrodialysis (ED) system, ion exchange membrane (IEM) avoids the limitation during the processes of the NF systems such as high concentration gradient and osmotic pressure.^[^ ^4^ ^]^ However, rapid development of biotechnologies with application of IEMs makes biofouling an actual problem.^[^ ^5^ ^]^ Different from organic and inorganic pollution, biological contamination is formed by the growth of bacteria on the surface with the potential to reproduction.^[^ ^6^ ^]^ Microbial‐mediated biological contaminants accumulated and forming biofilms, which increases the energy consumption and reduces the separation efficiency and their lifespan.^[^ ^7^ ^]^ Recent researches on anti‐biological contamination membranes were mainly focused on pressure membranes (i.e., MF, UF, NF, RO, etc.).^[^ ^8^ ^]^ Due to the constant addition of the feed solution (with the renewal of bacteria) in industrial ED process, the improvement of anti‐biological contamination performance is of vital importance to ensure the long‐term stability of IEMs.

It is reported that antibiotic resistance (ABR) has become a serious problem for industrial applications.^[^ ^9^ ^]^ Meantime, the bacteria in biofilm are 1000‐fold more resistant to normal antibiotics while comparing to single bacteria.^[^ ^9^ ^]^ Research on surfaces has been favored in recent years, but the antibacterial properties of single‐function surfaces fail with single or few applications.^[^ ^10^ ^]^ Nevertheless, the irreversible consumption and unsustainability are always the bottleneck that limits the anti‐biological stability of membrane.^[^ ^11^ ^]^ A rechargeable, multifunctional anti‐biological system should be explored to increase the long‐term sustainable utilization of IEMs.

It has been widely reported that most bacteria and the corresponding biofilms were negatively charged.^[^ ^12^ ^]^ The electrostatic attraction with the quaternary ammonium groups on AEMs caused an adhesion phenomenon of bacteria.^[^ ^8b^ ^]^ The bacteria eventually contaminated the surface of AEMs and formed a biofilm.^[^ ^13^ ^]^ To overcome this, “anti‐adhesion and antibacterial” by adjusting surface hydrophilicity and charge could enable a long‐term effective antibacterial surface.^[^ ^14^, ^15^ ^]^ Common antibacterial substances include lysozyme, capsaicin, heavy metals, their oxides, etc.^[^ ^16^ ^]^ Among them, cobalt and its alloys played an important role in the design of heavy metal antibacterial applications.^[^ ^17^ ^]^ Meanwhile, Co NP_S_ showing the potential of the highly antibacterial effect could be obtained from Co (II) via being reduced by NaBH_4_.^[^ ^18^ ^]^ N‐chloramine compounds, are more stable and less corrosive than free halogens, providing additional anti‐biofilm properties and reproducible antibacterial activity.^[^ ^7^, ^19^ ^]^

**Scheme 1:**
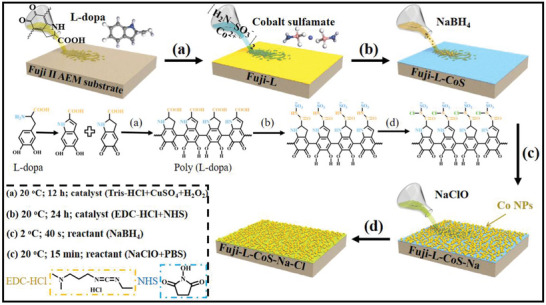
The process of AEMs surface modification: a) Fuji‐L; b) Fuji‐L‐CoS; c) Fuji‐L‐CoS‐Na, and d) Fuji‐L‐CoS‐Na‐Cl.

Encouraged by the above results of investigations, a synergistic antibacterial mechanism was proposed to enhance the hydrophilic and anti‐biofilm property of the membranes. Herein, mussels‐inspired polymerization was conducted and the amino groups of cobalt sulfamate were covalently incorporated on the membrane surface via the EDC‐HCl and NHS coupling reaction. Co (II) was reduced to Co NPs on the membrane surface to achieve a synergistic antibacterial performance (see the procedure in **Scheme** **1**). In addition, the negative charge on membrane surface renders an electrostatic repulsion effect on bacteria. The anti‐biofouling abilities of the membranes were analyzed qualitatively and quantitatively by adopting common Gram‐negative (*E. coli*) and Gram‐positive (*S. aureus & Bacillus*) bacteria as model biofouling organisms. Optimized membranes were evaluated for separation performance, energy consumption, and current efficiency in NaCl solution desalination process. In addition, MD simulations were performed using the LPS of *E. coli* with modified AEMs (see the chemical structures in Figure S1, Supporting Information) to reveal the interaction and adsorption energy. Considering the positive effect of the enhanced antibacterial surface, the fabrication strategy of AEMs with rechargeable anti‐biological performance could provide a valuable reference for the design of membrane materials resistant to biological contamination.

## 2 Results and Discussion

**Figure 1:**
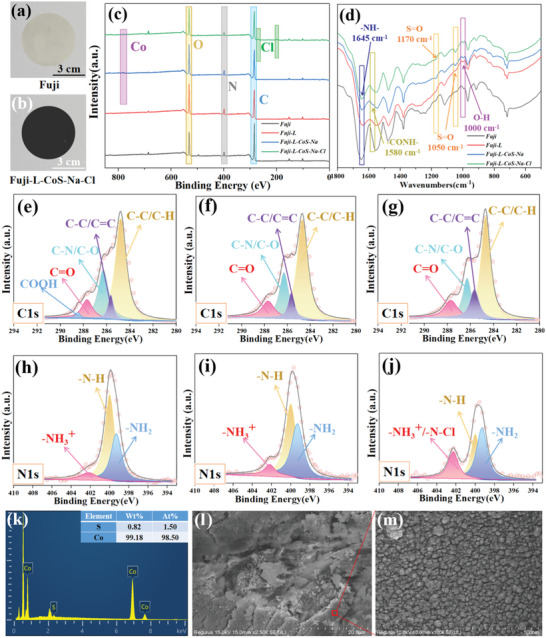
Photographs of the a) Fuji and b) Fuji‐L‐CoS‐Na‐Cl; XPS c) and ATR‐FTIR d) spectra of as‐prepared AEMs; C1s and N1s core level XPS spectra for as‐modified membranes: e,h) Fuji‐L, f,i) Fuji‐L‐CoS‐Na and g,j) Fuji‐L‐CoS‐Na‐Cl. EDX spectroscopy k) and SEM images l,m) of sodium sulfamate after reduction by NaBH_4_.

**Figure** **1**a,b shows the photographs of the original membrane and the Fuji‐L‐CoS‐Na‐Cl, respectively. The color of original membrane changes from grayish white to black during the modification process. Chemical structures of the as‐prepared membrane were characterized by XPS (Figure 1c) and ATR‐FTIR (Figure 1d) spectra. Binding energy peaks of Co and Cl element can be observed on the surface of Fuji‐L‐CoS‐Na‐Cl membrane. From ATR‐FTIR spectra, the peak at 1645 cm^−1^ represents the ─N─H─ bonds deformation vibration of amide. The ─N─H─ bonds in‐plane bending and ─N─C─ bonds stretching vibration of ─CO─NH─ groups fixed at 1580 cm^−1^ peak were appeared for Fuji‐L‐CoS‐Na and Fuji‐L‐CoS‐Na‐Cl membranes.^[^ ^20^ ^]^ Notably, the emerging characteristic peaks located at 1170 and 1050 cm^−1^ results from the symmetric stretching vibrations of ─S**═**O bonds and the symmetric and asymmetric stretching vibration of ─S─O─ bonds. The results confirm the presence of ─SO_3_H groups on Fuji‐L‐CoS‐Na and Fuji‐L‐CoS‐Na‐Cl membranes. In addition, the signals of stretching vibration (─O─H─ bonds) could be observable at 1000 cm^−1^ for Fuji‐L, Fuji‐L‐CoS‐Na, and Fuji‐L‐CoS‐Na‐Cl membranes.^[^ ^21^ ^]^ The XPS results of those samples were used to investigate the surface chemistry of membranes. The C1s spectra (Figure 1e–g) bears five distinct peaks of C─C/C─H (284.8 eV), C═C/C─C (285.8 eV), C─N/C─O (286.3 eV), C═O (287.7 eV) and COOH at 288.5 eV.^[^ ^22^ ^]^ The XPS spectra at 286.3 and 288.5 eV confirm the characteristic of L‐dopa for the modified membrane surface. Meanwhile, N1s spectra (Figure 1h–j) is fitted to ─NH_2_ (399.3 eV) bonds, N─H (400.0 eV) and ─NH_3_ **^+^** (402.2 eV) groups.^[^ ^23^ ^]^ Notably, the relative area of the peak at 402.2 eV (Figure 1j) is higher and the relative area of the peak at 400.0 eV was lower than that of the other membranes. The increase in peak area of 402.2 eV is due to the formation of N−Cl bonds. This is due to the higher electro‐negativity of Cl element than H.^[^ ^24^ ^]^ Moreover, Cl_2p_ spectrum (Figure S2, Supporting Information) is fitted to two components including the ─N─Cl (200.6 eV) bonds and Cl**^−^** (197.6 eV).^[^ ^7^, ^25^ ^]^ To ensure the reduction of cobalt sulfamate, EDX spectroscopy elemental analysis was used to test the S and Co content. Figure 1k shows that the ratio of S/Co has been greatly reduced compared to the element ratio (2:1) before reduction. Furthermore, SEM images of these samples are also shown in Figure 1l,m. Notably, Figure 1m shows the uniformly dispersed Co NPs (20–50 nm) on the conductive glue.

To further confirm the chemical composition of the modified membrane, atomic and mass percentage composition of six elements (C, N, O, S, Cl, and Co) were determined via EDX spectrum. The addition of cobalt sulfamate results in the increased content of O, S and Co element in Fuji‐L‐CoS (Figure S3b, Supporting Information) compared with that in Fuji‐L (Figure S3a, Supporting Information). For Fuji‐L‐CoS‐Na (Figure S3c, Supporting Information), the content of Co element is higher than that of Fuji‐L‐CoS, which is due to the reduction property of L‐dopa in NaBH_4_ solution.^[^ ^26^ ^]^ Correspondingly, the atomic and weight percentage of Co and S elements on the surface of as‐prepared AEMs were shown in Figure S3e,f (Supporting Information). Notably, the content of Cl element in Fuji‐L‐CoS‐Na‐Cl (Figure S3d, Supporting Information) is significantly increased while comparing to other modified membranes due to the formation of N─Cl bonds (Figure 1g_2_; Figure S2, Supporting Information). In addition, the strong oxidant (NaClO solution) does not obviously affect the content of S and Co elements.

**Figure 2:**
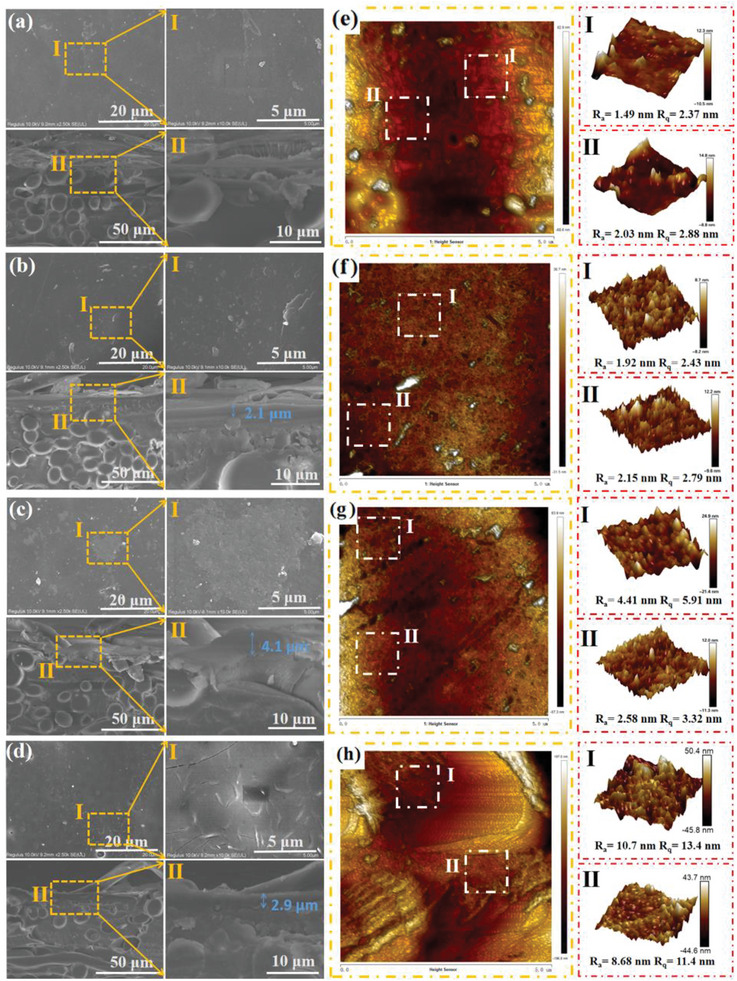
SEM images of as‐prepared AEMs surface and cross‐section: a) Fuji, b) Fuji‐L, c) Fuji‐L‐CoS‐Na, and d) Fuji‐L‐CoS‐Na‐Cl; AFM images of as‐prepared AEMs surface: e) Fuji, f) Fuji‐L, g) Fuji‐L‐CoS‐Na, and h) Fuji‐L‐CoS‐Na‐Cl.

The SEM images before and after surface modification are showed in **Figure** **2** and Figure S4 (Supporting Information). Fuji membrane (Figure 2aI) exhibits a smooth and flat surface and obvious fibrous support materials was shown at the cross‐section (Figure 2aII). The flake‐like accumulation on the surface of Fuji‐L membrane (Figure 2bI) is due to the oxidative polymerization of L‐dopa. Meanwhile, its cross‐section (Figure 2bII) has an increased thickness compared to the substrate. After chemical assembly of cobalt sulfamate, the surface of Fuji‐L‐CoS‐Na (Figure 2cI) become rougher as Co NPs was generated. In addition, the membrane without the Co NPs in Figure S4 (Supporting Information) showed a lower roughness than Fuji‐L‐CoS‐Na. The cobalt deposited on Fuji‐L‐CoS‐Na membrane surface is more densely compared to Fuji‐L‐CoS. The cross‐section of Fuji‐L‐CoS‐Na (Figure 2cII) is different from the cross‐section of Fuji membrane. In comparison with the substrate, the increased thickness of the cross‐section (Figure 2cII) is particularly pronounced. The oxidized membrane (Figure 2dI) is rougher than other membranes. Herein, surface roughness of different membranes is tested by AFM. As shown in Figure S5a (Supporting Information), the roughness of the membranes increases. Comparing to the membranes after surface modification, Fuji membrane (Figure 2e) exhibits the lowest surface roughness. The roughness of the membranes does not exhibit a significantly increase after the oxidative self‐polymerization of L‐dopa (Figure 2f) and chemical assembly of cobalt sulfamate (Figure S5b, Supporting Information). However, the AFM images show that Fuji‐L and Fuji‐L‐CoS (Figure S5b, Supporting Information) have more protrusions compared to that of the Fuji membrane. Correspondingly, the SEM images of Fuji‐L and Fuji‐L‐CoS are rougher compared to the original membrane. The roughness was shown in Figure 2g after the reduction of NaBH_4_ increases, which is due to the transition process from Co (II) to Co NPs. Because of the strong oxidizing (NaClO) solution, Fuji‐L‐CoS‐Na‐Cl surface possesses the largest roughness (Figure 2h) value of these membranes.

**Figure 3:**
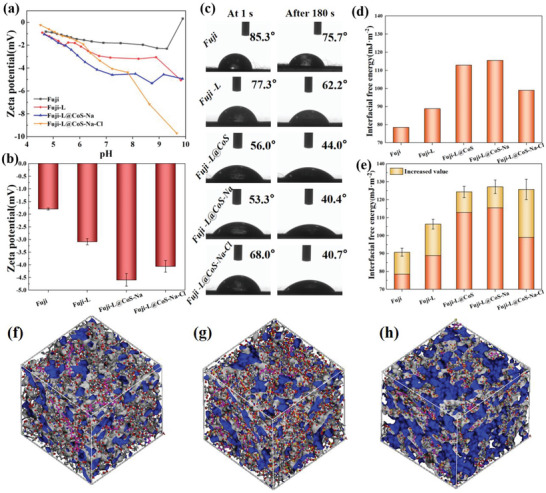
a) Zeta potential of membranes with pH at room temperature; b) The zeta potential value of the membrane at pH 7.0 ± 0.2; c) The photographs of a water droplet on membranes and that of after 3 min at room temperature; d) The interfacial free energy of the as‐prepared membranes at 25 °C; e) The increased value of AEMs interfacial free energy after 180 s. Simulated free volume of f) Fuji‐L, g) Fuji‐L‐CoS‐Na, and h) Fuji‐L‐CoS‐Na‐Cl surface modification layer.

**Figure** **3**a shows the zeta potential curves of as‐prepared membranes at pH range of 4–10. Accordingly, Figure 3b shows the zeta potential value of the membrane at pH 7.0 ± 0.2. It is seen that the zeta potential value of modified is lower than that of the substrate, which can be possibly due to the assembly of carboxyl and sulfonate groups on the surface of modified membrane. Also, the hydrophilicity of the surface‐modified membrane has also been evaluated in terms of water contact angles (WCA). As is seen from Figure 3c that the WCA values of the samples is inversely proportional to the hydrophilicity and the value of modified membrane is lower than that of the substrate membrane. In addition, the liquid drop could spread on the Fuji‐L‐CoS‐Na‐Cl after 180 s, indicating the hydrophilicity performance.^[^ ^27^ ^]^ To further confirm this, the membrane‐liquid interfacial free energy was tested. As it shown in Figure 3d, the value is proportional to the surface hydrophilicity.^[^ ^28^ ^]^ Interestingly, the increased value of Fuji‐L‐CoS‐Na‐Cl membrane‐liquid interfacial free energy is the largest among the five membranes (Figure 3e). The possible reason is that the instant of contact with water drops will trigger the reversible reaction of N─Cl bonds.^[^ ^19b^ ^]^ Subsequently, fractional free volume (FFV) of Fuji‐L, Fuji‐L‐CoS‐Na, and Fuji‐L‐CoS‐Na‐Cl surface modification layer were simulated to explain this phenomenon. The FFV of Fuji‐L (Figure 3f), Fuji‐L‐CoS‐Na (Figure 3g), and Fuji‐L‐CoS‐Na‐Cl (Figure 3h) are 21.90%, 22.37%, and 50.21%, respectively. Compared to the two layers (Fuji‐L and Fuji‐L‐CoS‐Na), the maximum FFV of Fuji‐L‐CoS‐Na‐Cl can be explained by the formation of N─Cl bonds (Disruption of hydrogen bonds between the backbone of molecules). Correspondingly, the increase of FFV and surface roughness (Figure 2h) is conducive to water penetration and microbial contact. It is speculated that N‐chloramine compounds can expand the molecular main chain spacing of the original coating, resulting in a formation of free volume.

IEMs will swell in DI water due to the presence of hydrophility of ion exchange groups. **Table** **1** lists the SR (length and thickness) values of as‐prepared membranes. As it shown, it is seen that the SR values of the membranes after modification almost do not changes, due to the substrate's fibrous (Figure 2a). Generally, the IEC values of AEMs decreases during the modification process. Meanwhile, Fuji‐L‐CoS‐Na has the smallest IEC values among the five as‐tested membranes. Because Co NPs may affect the functional location of membrane during reduction process, as shown in Figure 2cI. Furthermore, the difference in transport numbers of as‐modified membranes is <2% compared with the original membrane. The desalination performance of the 0.5 m NaCl solution for Fuji, Fuji‐L, Fuji‐L‐CoS‐Na, and Fuji‐L‐CoS‐Na‐Cl membranes is shown in Figure S6 (Supporting Information). The difference in desalination rate of the four membranes during 240 min (three cycles; Figure S6a–c, Supporting Information) was small. Compared to the pre‐desalted membranes, the differences in color, energy consumption, and current efficiency (Figure S6e,d, Supporting Information) of the desalted membranes were not significant. To further explore the stability of cobalt sulfamate and Co NPs, the energy‐dispersive X‐ray spectroscopy elemental maps of S and Co on the Fuji, Fuji‐L‐CoS‐Na, and Fuji‐L‐CoS‐Na‐Cl membrane surface (original and after three‐cycle ED desalination) were shown at Figure S7 (Supporting Information). The S and Co element content of the original and as‐modified membranes after three‐cycle ED desalination is similar to the pristine. The color and the EDX map of the membranes can synergistically demonstrate the stability of the modified layer.

**Table 1:** The SR (length and thickness), IEC and mean ions transport number values of as‐prepared membranes.

Samples	SR‐length [%]	SR‐Thickness [%]	IEC [mmol g^−1^]	Mean ions transport number
Fuji	3.19 ± 0.03	8.3 ± 0.7	1.52 ± 0.03	a
Fuji‐L	2.97 ± 0.02	8.0 ± 0.3	1.53 ± 0.03	a ± 1.09%a
Fuji‐L‐CoS	3.27 ± 0.04	11.7 ± 0.9	1.43 ± 0.03	a ± 1.71%a
Fuji‐L‐CoS‐Na	1.44 ± 0.02	9.9 ± 0.9	1.40 ± 0.02	a ± 1.55%a
Fuji‐L‐CoS‐Na‐Cl	3.32 ± 0.04	11.37 ± 0.3	1.47 ± 0.08	a ± 1.09%a

**Figure 4:**
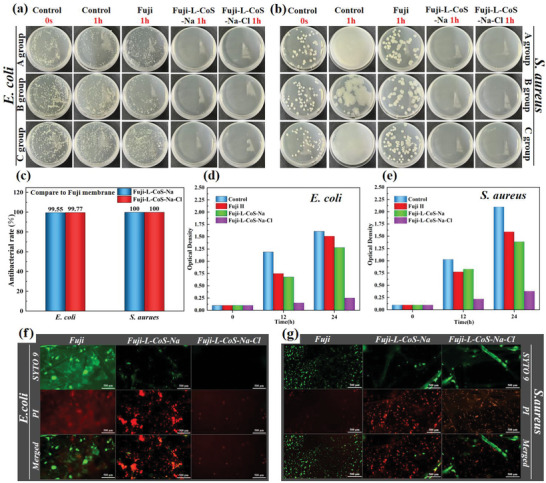
The antibacterial performance of a) *E. coli* and b) *S. aureus* was measured by ASTM E2149 method for control group (0 s and 1 h), Fuji (1 h), Fuji‐L‐CoS‐Na (1 h) and Fuji‐L‐CoS‐Na‐Cl (1 h); c) The antibacterial rate of Fuji‐L‐CoS‐Na and Fuji‐L‐CoS‐Na‐Cl membrane (*E. coli* and *S. aureus* was measured by ASTM E2149 method); d) *E. coli* and e) *S. aureus* were selected for antibacterial tests on OD values of the control group, original membrane, Fuji‐L‐CoS‐Na and Fuji‐L‐CoS‐Na‐Cl; Fluorescence microscopy images of the f) *E. coli* and g) *S. aureus* on the surface of Fuji, Fuji‐L‐CoS‐Na, and Fuji‐L‐CoS‐Na‐Cl.

**Figure 5:**
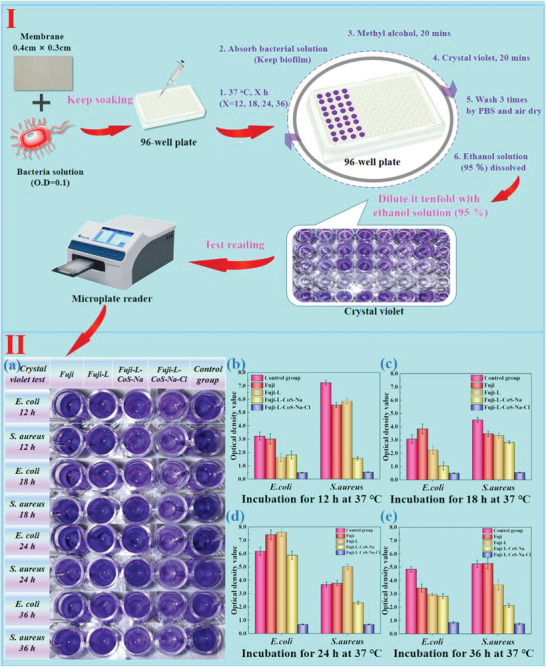
I) The process of anti‐biofilm operation via crystal violet; (II) a) Photographs of the crystal violet tests on those groups; Evaluation of anti‐biofilm effects using control, Fuji, Fuji‐L, Fuji‐L‐CoS‐Na, and Fuji‐L‐CoS‐Na‐Cl groups against *S. aureus* and *E. coli* biofilm within b) 12, c) 18, d) 24, and e) 36 h.

The bacteria and microorganisms in solution will form bacterial colloids, leading to the increased viscosity. Therefore, it is necessary to solve the problem of bacterial/microbial adsorption on membrane surface. Here, ASTM E2149 method was used to test the antibacterial performance of AEMs before and after modification (**Figure** **4**a–c; Tables S2 and S3, Supporting Information). Since the difference in antibacterial ratio between the control group and the Fuji membrane was >15%, the antibacterial performance of Fuji‐L‐CoS‐Na and Fuji‐L‐CoS‐Na‐Cl membrane should be compared with the original substrate. Colony‐forming units per milliliter (CFU/mL) can be deduced back from the average colony count, and Fuji‐L‐CoS‐Na and Fuji‐L‐CoS‐Na‐Cl membrane have high antibacterial performance on both *E. coli* and *S. aureus* (>99%). Furthermore, the antibacterial test of the bacterial eluate (*E. coli* and *S. aureus*) is shown in Figure 4d,e. Its OD results deviate slightly from that of ASTM method, because the concentration of bacteria is higher, and quantification is more difficult. Furthermore, part of the live bacteria adhering to the membrane surface is difficult to wash down and this phenomenon will cause inaccuracy of antibacterial results. The possible reason was that Co NPs was firmly fixed on the membrane surface and did not spread into the bacterial solution. In fact, it is more accurate to study the state of the bacteria on the membrane surface than the state of bacterial eluate. Fluorescence microscopy therefore was used to measure the antibacterial activity of Fuji, Fuji‐L‐CoS‐Na, and Fuji‐L‐CoS‐Na‐Cl (Figure 4f,g), the red areas (PI dyes) are dead bacteria and the green areas (SYTO 9 dyes) are live bacteria.^[^ ^29^ ^]^ In addition, both dyes are also absorbed by the fibers of substrate and appear red or green in the images. The *S. aureus* and *E. coli* have a low mortality rate on the surface of Fuji membranes. This fraction of live bacteria can multiply and form biofilms on the surface of membrane, affecting the performance of the AEMs. In contrast, the mortality rate of *S. aureus* on the surface of Fuji‐L‐CoS‐Na membrane could reach 96.84% (ImageJ manual counting). The antibacterial enhancement is significant compared to the substrate. Due to the released Co (II) from Co NPs, which interacts with the sulfhydryl groups of the basic bacterial enzymes and the bacteria die.^[^ ^18^ ^]^ For the Fuji‐L‐CoS‐Na‐Cl (*E. coli* group), its antibacterial properties were significantly higher than other two membranes. In addition to Co NPs, N‐chloramine compounds (N─Cl bonds) have also been improved to the membrane surface with antibacterial properties when released in water or other solutions. Furthermore, the bacterial density of Fuji‐L‐CoS‐Na‐Cl membrane (*E. coli* group) was lower than that of the other groups. The reason we speculate is that the release of Cl element will improve anti‐adhesion performance. The quantitative anti‐biofilm tests of the original and as‐modified membranes were studied via crystal violet method. **Figure** **5**I shows the whole process of anti‐biofilm operation and Figure 5II shows the results of crystal violet in control and as‐prepared membrane groups. Generally, the O.D values and photographs (Figure 5IIa) of control, Fuji and Fuji‐L groups were similar at 12 h (Figure 5IIb), 18 h (Figure 5IIc), 24 h (Figure 5IId), and 36 h (Figure 5IIe). Meanwhile, the O.D value of Fuji‐L‐CoS‐Na group increases at 24 h in *E. coli* solution. Furthermore, For the Fuji‐L‐CoS‐Na‐Cl membrane group, the O.D value of biofilm (crystal violet) have slightly change. This is because N‐chloramine compounds have this performance to anti‐biofilm formation. The results indicated that the anti‐biofilm ability of Fuji‐L‐CoS‐Na‐Cl was better than other groups, which also indicated that the combination of Co NPs and N‐Cl bond had synergistic anti‐biofilm properties.

**Figure 6:**
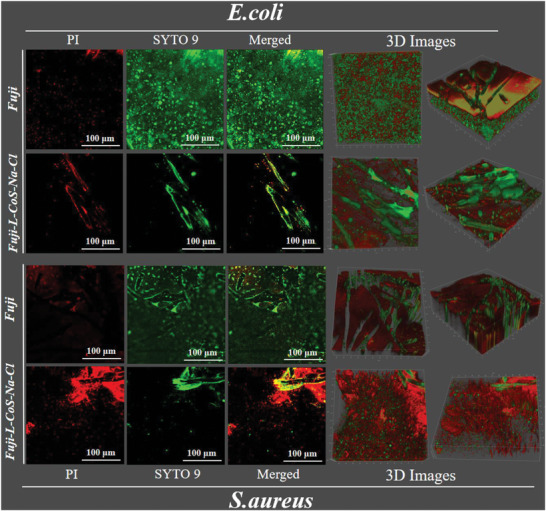
CLSM images show the viability of *S. aureus* and *E. coli* biofilms after they were incubated with Fuji and Fuji‐L‐CoS‐Na‐Cl membrane for 18 h.

CLSM was used to observe the distribution of live and dead bacteria in the biofilm on the surface of Fuji and Fuji‐L‐CoS‐Na‐Cl (**Figure** **6**). The sectional diagram and 3D CLSM images show live bacteria fluorescent green (SYTO9 dyes) and dead bacteria fluorescent red (PI dyes). Red fluorescence can be observed in all tested groups. For the substrate membrane, binding of quaternary ammonium salts to bacteria leads to bacterial death.^[^ ^30^ ^]^ In addition, the number of live bacteria was greater than the dead bacteria and the increase in biofilm thickness could be seen in the CLSM 3D images. Consistent with the results presented by fluorescence microscopy (Figure 4), this indicates that the original membrane does not possess long‐term antibacterial and anti‐biofilm properties. For the Fuji‐L‐CoS‐Na‐Cl, *E. coli* group showed less biofilm formation on the surface of membrane and showed fluorescent red (Except for the substrate membranes, which were fibrillar fluorescent green). *S. aureus* showed a stronger ability to reproduce (Biofilm formation capacity) than *E. coli* here and the biofilms were consistent with the anti‐biofouling results observed by fluorescence microscopy correspondingly. Nevertheless, negatively charged bacteria were electrostatically repelled (─SO_3_ **^−^** groups) close to the surface of Fuji‐L‐CoS‐Na‐Cl. Furthermore, the Cl element on the as‐prepared layer (release of the N─Cl bond) also repels bacteria via liquid‐membrane interaction forces. Bacterial adsorption needs to provide higher energy due to the increased interfacial free energy (hydrophilicity performance) on the surface of membrane. The enhancement of the anti‐biofilm formation properties was attributed to the oxidation of N─Cl bonds and the contact of Co NPs.

**Figure 7:**
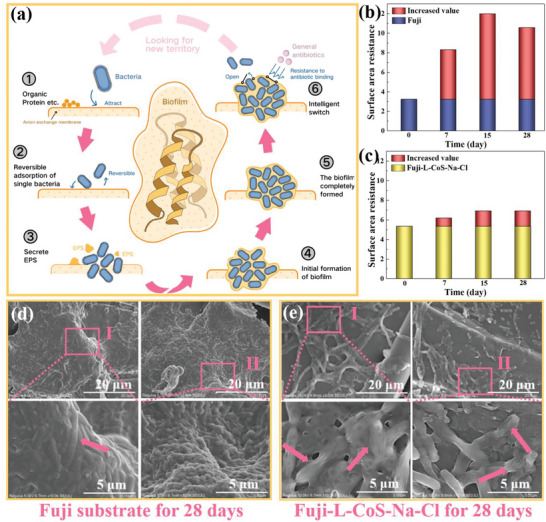
a) The growth cycle of biofilms on anion exchange membranes; The increased surface area resistance of b) Fuji and c) Fuji‐L‐CoS‐Na‐Cl after they soaked in the bacterial solution for 0,7, 15, 28 d; SEM images of d) Fuji and e) Fuji‐L‐CoS‐Na‐Cl membrane after they soaked in the bacterial solution for 28 d.

Generally, biofilms are synthesized by a high density of microorganisms encased in a hydrated matrix.^[^ ^31^ ^]^ It is also a dynamic system with a complex structure that can colonize both biotic and abiotic surfaces.^[^ ^31^, ^32^ ^]^ IEMs can provide good carriers for the formation of biofilms, which has become a practical problem affecting the operation of IEMs.^[^ ^5^, ^7^ ^]^ **Figure** **7**a shows the whole process of biofilm formation on the surface of AEMs.^[^ ^33^ ^]^ In the first and second stages, bacteria reversibly attached to the AEM surface. In the third and fourth stages, the EPS matrix is secreted by bacteria and the biofilm is initially formed. In the final two stages, some bacteria can be separated from the mature biofilm and dispersed in different locations (on the AEMs surface) to form new biofilms. Therefore, it is necessary to investigate the long‐term anti‐biofilm performance for AEMs.

Here, we investigated the anti‐biofilm effect of the Fuji substrate and Fuji‐L‐CoS‐Na‐Cl over 28 d. It can be seen from Figure 7b,c that the surface area resistance of the Fuji and Fuji‐L‐CoS‐Na‐Cl increases with time. Meanwhile, it is difficult to quantitatively evaluate the degree of biofilm contamination during the biofouling process.^[^ ^5^ ^]^ In order to simulate the real phenomenon of biological contamination, the mixed Gram‐positive (*S. aureus*) and Gram‐negative (*E. coli*) were selected. The thickness of the bacteria increased significantly after 28 days contamination (Fuji substrate, Figure 7d). Especially in Figure 7dI, the biofilm was mature, and the bacteria were intact (Indicated by arrow). Moreover, biofilms in this state had the ability to protect the bacteria and were difficult to bind to antibiotics in general.^[^ ^34^ ^]^ Compared to substrate, Fuji‐L‐CoS‐Na‐Cl (Figure 7e) could inhibit biofilm formation to a great extent. This is due to long‐term effects of Co NPs and the oxidation of N‐chloramine compounds. Moreover, the bacteria appear shriveled and incomplete (as indicated by the arrow) due to Co NPs and N─Cl bonds.

**Figure 8:**
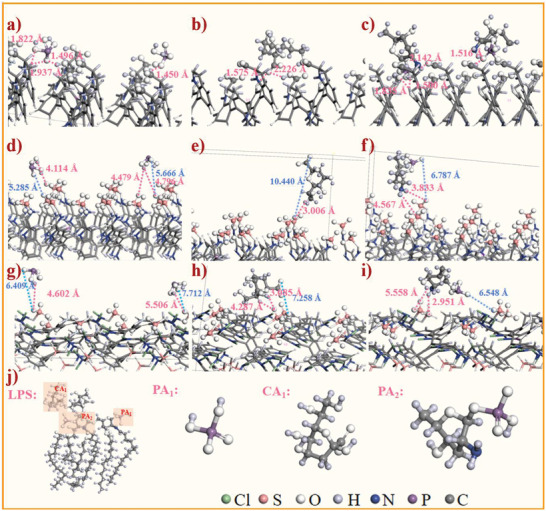
Primary hydrogen bonds of a) PA_1_, b) CA_1_, c) PA_2_ on the surface of Fuji‐L layer; Primary hydrogen bonds of d) PA_1_, e) CA_1_, f) PA_2_ on the surface of Fuji‐L‐CoS‐Na layer; Primary hydrogen bonds of g) PA_1_, h) CA_1_, i) PA_2_ on the surface of Fuji‐L‐CoS‐Na‐Cl layer; j) The chemical structure of *E. coli* LPS, PA_1_, CA_1_, and PA_2_. The units of hydrogen bonds are Å.

LPS, as the main component of the outer membrane (OM) of *E. coli* (Figure S8, Supporting Information), the according structures and fragments (LPS_1_ and LPS_2_) of LPS are shown in Figure S9 (Supporting Information). The average adsorption energy (Figure S10a, Supporting Information) of the three modified layers gradually increased with the average loading (Figure S10b, Supporting Information) gradually decreased. Adsorption loading of Fuji‐L is the largest in the three modified layers and the FFV gradually increases due to spatial site resistance (Figure 3f–h). However, the adsorption of LPS and its two fragments remained reduced, which was explained by stronger electrostatic. Considering the complex configuration of LPS, the interaction between its partially simplified structures (PA_1_, CA_1_, and PA_2_, **Figure** **8**j) and the modification layers (Fuji‐L, Fuji‐L‐CoS‐Na, and Fuji‐L‐CoS‐Na‐Cl). PA_1_ (Figure 8a), CA_1_ (Figure 8b), and PA_2_ (Figure 8c) all have van der Waals force and form strong hydrogen bonds to Fuji‐L. Among them, the main hydrogen bonds of H_2_PO_4_ **^−^** (PA_1_) with a minimum bond length of 1.450 Å (red line). In contrast, Fuji‐L‐CoS‐Na (Figure 8d–f) and Fuji‐L‐CoS‐Na‐Cl (Figure 8g–i) have weak hydrogen bonds (**>**2.5 Å) due to electrostatic repulsion (Blue line). Furthermore, the dynamic free diffusion process of 20 PA_1_ on three modified layers surface was further selected (Video S1, Supporting Information). The movement trajectories (Distance of the modified layer) of these PA_1_ within 50 ps are shown in Figure S10c–e (Supporting Information). It was evident that >30% of PA_1_ is adsorbed by the surface of Fuji‐L layer, and this portion of PA_1_ gradually approach the modified layer to form hydrogen bonds. The trajectories of PA_1_ in Fuji‐L‐CoS‐Na and Fuji‐L‐CoS‐Na‐Cl showed that all ions gradually moved away from the modified layer, suggesting a repulsive effect of the modified layer.

## 3 Conclusion

A rechargeable multifunctional anti‐biological system is reported that is attached to the AEM surface though a mussel‐inspired L‐dopa connection strategy. Co NPs and N‐chloramine compounds were imparted on the AEM surface via a two‐step modification to demonstrate the “anti‐adhesion and anti‐biofilm” mechanism. The anti‐biofilm and antibacterial abilities of the substrate and optimal‐modified membranes were qualitatively and quantitatively studied. The biological resistance of modified AEM significantly improves and the optimized membrane exhibits no sacrifice concerning the NaCl desalination properties. In addition, LPS from *E. coli* has been used for MD simulation to confirm the interaction, adsorption energy and average loading. The as‐prepared AEM with rechargeable anti‐biological performance could provide a valuable reference for the designation of novel materials with biological contamination resistant abilities.

## 4 Experimental Section

### Materials and Chemicals

1‐Ethyl‐3‐(3‐dimethylaminopropyl) carbodiimide hydrochloride (EDC‐HCl, 98.5%), N‐hydroxysuccinimide (NHS, 98%), sodium dihydrogen phosphate (NaH_2_PO_4_, 99%), cobalt sulfamate (≥98%), disodium hydrogen phosphate (Na_2_HPO_4_, 99%), tris(hydroxymethyl)aminomethane (Tris, 99.5%), and crystal violet (AR) were purchased from Shanghai Maclin Biochemical Technology Co., LTD. L‐dopa (99%), sodium hypochlorite solution (NaClO, >5%), sodium borohydride (NaBH_4_, AR) sodium chloride (NaCl, AR), sodium sulfate (Na_2_SO_4_, AR), hydrochloric acid (HCl, AR), sodium hydroxide (NaOH, AR), copper sulfate(CuSO_4_, 99%), hydrogen peroxide (H_2_O_2_, ≥30%, AR), tryptone (LP0042), yeast powder (LP0021), AGAR powder (A505255‐0250), methyl alcohol (AR), acetic acid (AR), propidium iodide (PI, ≥94.0%), glutaraldehyde (50% in water, AR), ethanol (75%, AR), and ethanol (95%, AR) were purchased Shanghai Aladdin Reagent Co., LTD. SYTO9 and PI dyes (LIVE/DEAD BacLight Bacterial Viability Kit) were purchased Thermo Fisher Scientific Inc. All the other reagents and solvents were bought from commercial sources and used as received without further purification. Distilled water (DI water) was used throughout the whole experiment. The strains used in this experiment were *E. coli* (K88), *S. aureus* (ATCC25923), and Bacillus (CCTCC AB 90 008).

### Membrane Modification

Pretreatment was necessary, chemical immersion (0.2 m NaOH, 0.5 m NaCl, and DI water for 0.5, 0.5, and 24 h, respectively) of the membrane was required before modification. The modification process (include time and temperature of reaction) of the surface is shown in Scheme 1: a) L‐dopa (10.01 mm) dissolved in Tris‐buffer solution (pH 8.5, 50 mm, 100 mL) oxidation self‐polymerization on the membrane surface via CuSO_4_ and H_2_O_2_ as the trigger;^[^ ^21^, ^35^ ^]^ b) The amino group of cobalt sulfamate (17.5 mm) was covalently incorporated on the membrane surface of carboxyl via the EDC‐HCl (4.1 mm) and NHS (7 mm) coupling reaction in DI water (100 mL);^[^ ^36^ ^]^ c) Co (II) was reduced to Co NPs attached to the membrane surface via NaBH_4_ (10 mm, 100 mL of DI water solution);^[^ ^37^ ^]^ d) The N─H bond was converted to N─Cl by exposing to NaClO solution (diluted ten‐fold by PBS) via N‐chloramination reaction.^[^ ^19b^ ^]^

### Membrane Characterizations

Detailed instrumental analysis information for X‐ray photoelectron spectroscopy (XPS), Attenuated total Reflection‐Fourier transform infrared (ATR‐FTIR), scanning electron microscope (SEM), energy dispersive X‐Ray spectroscopy (EDX), and atomic force microscopy (AFM) are available in Supporting Information. Zeta potential analyzer (SurPASS3, Anton Paar, Austria) for was used to test the potentials of membranes surface by a streaming potential method in a 1.0 mm KCl electrolyte. Tests on water contact angle analyzer (WCA, Dataphyscis, Germany) were carried out on five different points on surface of as‐prepared membranes, and an average value was calculated. The membrane surface interface free energy (‐ΔGSL)^[^ ^28^, ^38^ ^]^ is calculated according to −ΔGSL = γL(1+cosθ/Δ) where *θ* is the membrane surface average water contact angle, γL is the liquid surface tension (72.8 mJ m^−2^ for water at 25 °C), Δ = actual surface area divided by the planar area. The actual surface area and planar area are recorded by AFM. Tests of ion exchange capacity (IEC), swelling ratio (SR) and surface area resistance are available in Supporting Information.

### ED Desalination

To measure the separation performance of as‐prepared AEMs by surface modification, ED desalination was carried out at lab‐made device. The lab‐made device and experimental methods were described in previous studies.^[^ ^39^ ^]^ Before testing, 80 mL NaCl (0.5 m) solution was filled in the two middle cell and pump the Na_2_SO_4_ (0.3 m) solution into the electrode chamber (keep circulating). The conductivity of the dilute and concentrate compartments were measured by a conductivity meter every 10 min. The volumes of the concentration and dilute cells were measured when the ED process was completed. The details of current efficiency and energy consumption are described in Supporting Information.

### Antibacterial Test

Optical density test at bacterial eluent methods and operation process are described in Supporting Information. ASTM E2149 methods and operation process are also described in Supporting Information. Fluorescence microscopy (Axioscope 5, Zeiss, USA) was used to determine the antibacterial rate of AEMs. 100 µL *E. coli* (O.D = 0.100, dilute by PBS1) and *S. aureus* (O.D = 0.100, dilute by PBS1) was absorbed onto the membrane surface at 37 °C for 1 h (It was same to the “culture plates method” and described in Supporting Information). SYTO9 and PI dyes were added into the *S. aureus* and *E. coli* system. In details, the mixture solution samples were dyed away from light for ≈10 min. After mixture solution samples preparation were complete, fluorescence microscopy was used to distinguish live or dead bacteria. The anti‐biofilms properties of AEMs against *S. aureus* and *E. coli* was confirmed by crystal violet staining. *E. coli* (O.D = 0.100, dilute by liquid LB) and *S. aureus* (O.D = 0.100, dilute by liquid LB) were used for this study, the membrane contact bacterial solution were in a 96‐well plate for 12, 18, 24, and 36 h. Biofilm formation was checked via crystal violet staining test and O.D (Synergy LX, BioTek, USA, UV at 600 nm) value was recorded after they contacted. The experimental operation process is described in Supporting Information. Confocal laser scanning microscope (CLSM, Axioscope 5, Zeiss, American) was used to determine the strength of the anti‐biofilm. 2000.0 µL *E. coli* (O.D = 0.100, dilute by liquid LB) and *S. aureus* (O.D = 0.100, dilute by liquid LB) via contact with as‐prepared AEMs (1.5 cm **×** 1.5 cm) in 12‐well plate at 37 °C for 18 h. SYTO9 and PI dyes were added into the *S. aureus* and *E. coli* system. The mixture solution samples were dyed away from light for ≈30 min. After preparing the mixture solution samples, CLSM was used to determine the 3D morphology of biofilm on the surface of AEMs. The morphology of biofilms on the Fuji and Fuji‐L‐CoS‐Na‐Cl surfaces was monitored via SEM images (SU8100, Hitachi, Japan). *E. coli* (O.D = 0.100, dilute by liquid LB), *Bacillus* (O.D = 0.100, dilute by liquid LB) and *S. aureus* (O.D = 0.100, dilute by liquid LB) were used. The bacterial solution infiltrate Fuji II (*E. coli* and *S. aureus*) and Fuji‐L‐CoS‐Na‐Cl (*E. coli* and *Bacillus*) in the 12‐well plate. Those samples were soaked for 7, 15, and 28 d and then rinsed with sterile water three times. Then, soaked in glutaraldehyde (2.5%) buffer for 3 h at 4 °C and dried for 24 h at 40 °C in a vacuum oven. Furthermore, the membranes were recharged in NaClO solution for 10 min every 72 h. Correspondingly, the liquid LB and bacteria solution were replaced every 72 h.

### MD Simulations

Molecular dynamics (MD) simulations were built by the Forcite module of the Materials Studio software package (Accelrys). Here, the free volumes of Fuji‐L, Fuji‐L‐CoS‐Na, and Fuji‐L‐CoS‐Na‐Cl surface modification layer were simulated. After constructing the amorphous cells, the cell was optimized (Temperature: 298 K, Quality: ultra‐fine, Max iterations: 500, Forcefield: COMPASSII, Electrostatic: Ewald). Then, annealing optimization (Quality: ultra‐fine, Annealing cycles:5, Initial temperature 300 K, Mid‐cycle temperature 700 K, Heating ramps per cycle: 5, Dynamics steps per ramp:1000, Ensemble: NVE, Initial velocities: random). The model of LPS was built with the CHARMM‐GUI Membrane Builder.^[^ ^40^ ^]^ MD simulations were conducted to get an insight into the dynamics of the interaction between LPS (three fragments) and the modified layers (Fuji‐L, Fuji‐L‐CoS‐Na, and Fuji‐L‐CoS‐Na‐Cl). This simulation was operated in Materials Studio 2019, Amorphous Cell Forcite and Sorption modules were used for the system construction and calculation. The Sorption module was used to simulation adsorption of optimized cells (Establish vacuum layer 20 Å, the modified layers: Fuji‐L, Fuji‐L‐CoS‐Na, and Fuji‐L‐CoS‐Na‐Cl), and the adsorbates are LPS and its fragments. In addition, to explore the adsorption process of H_2_PO_4_ **^−^** (one of LPS fragment) by the modified layer (Fuji‐L, Fuji‐L‐CoS‐Na, and Fuji‐L‐CoS‐Na‐Cl), the dynamic processes of modified layer and adsorbed layer were established. The adsorbed layer was composed of 20 H_2_PO_4_ **^−^**. Both modified and adsorption layer were set to 40 Å in the *X*‐axis and 20 Å *Y*‐axis directions (length and width). Dynamic parameter: temperature: 298 K, quality: ultra‐fine, energy forcefield: COMPASSII, ensemble: NVT, initial velocities: random, time step: 1.0 fs, total simulation time: 25.0 ps, thermostat: nose. A Perl Script document was written to investigate the atoms trajectory in the *Z*‐axis direction. The trajectory of the 20 H_2_PO_4_ **^−^** is obtained (the distance from the modifier layer) in this process.

## Conflict of Interest

The authors declare no conflict of interest.

## Supporting information

Supporting Information

Supplemental Video 1

## Data Availability

The data that support the findings of this study are available in the supplementary material of this article.

## References

[1] a) L. Fan , Y. Ji , G. Wang , J. Chen , K. Chen , X. Liu , Z. Wen , J. Am. Chem. Soc. 2022, 144, 16; 10.1021/jacs.1c13740 35404594

[2] a) M. Shannon , P. Bohn , M. Elimelech , J. G. Georgiadis , B. J. Mariñas , A. M. Mayes , Nature 2008, 452, 301; 18354474 10.1038/nature06599

[3] a) C. Striemer , T. Gaborski , J. McGrath , P. M. Fauchet , Nature 2007, 445, 749; 17301789 10.1038/nature05532

[4] a) Y. Zhao , N. Mamrol , W. A. Tarpeh , X. Yang , C. Gao , B. Van der Bruggen , Prog. Mater. Sci 2022, 128, 100958;

[5] S. Mikhaylin , L. Bazinet , Adv. Colloid Interface Sci. 2016, 229, 34. 26813627 10.1016/j.cis.2015.12.006

[6] a) M. Herzberg , S. Pandit , M. S. Mauter , Y. Oren , J. Membr. Sci. 2020, 596, 117564;

[7] a) R. Ma , X. Lu , C. Wu , S. Zhang , S. Zheng , K. Ren , J. Gu , H. Wang , H. Shen , J. Membr. Sci. 2022, 660, 120886;

[8] a) J. Zhu , J. Wang , J. Hou , Y. Zhang , J. Liu , B. Van der Bruggen , J. Mater. Chem. A 2017, 5, 6776;

[9] a) R. C. MacLean , A. S. Millan , Science 2019, 365, 1082;31515374

[10] a) C. Su , Y. Ye , H. Qiu , Y. Zhu , ACS Appl. Mater. Interfaces 2021, 13, 10553; 33617220 10.1021/acsami.0c20033

[11] M. Yi , T. D. Nguyen , H. Liu , Y. Liu , S. Xiong , Y. Wang , Adv. Funct. Mater. 2023, 33, 2213471.

[12] a) A. Diaconu , T. Coenye , M. Barboiu , S. P. VincenT , Angew. Chem., Int. Ed. 2021, 60, 22505; 10.1002/anie.202109518 34346553

[13] Y. Wang , J. Wu , D. Zhang , F. Chen , P. Fan , M. Zhong , S. Xiao , Y. Chang , X. Gong , J. Yang , J. Zheng , J. Mater. Chem. B 2019,7, 5762. 31465075 10.1039/c9tb01313j

[14] a) T. Wei , Z. Tang , Q. Yu , H. Chen , ACS Appl. Mater. Interfaces 2017, 43, 37511; 10.1021/acsami.7b13565 28992417

[15] a) L. Zhang , J. Sha , R. Chen , Q. Liu , J. Liu , J. Yu , H. Zhang , C. Lin , W. Zhou , J. Wang , Appl. Catal. B 2020, 271, 118920;

[16] a) Y. Yuan , Y. Liu , Y. He , B. Zhang , L. Zhao , S. Tian , Q. Wang , S. Chen , Z. Li , S. Liang , G. Hou , B. Liu , Y. Li , Biomaterials 2022, 287, 121613; 35700621 10.1016/j.biomaterials.2022.121613

[17] a) Y. Bai , C. Shi , X. Ma , J. Li , S. Chen , N. Guo , X. Yu , C. Yang , Z. Zhang , Chem. Eng. J. 2022, 447, 137545.

[18] N. S. Alahmadi , J. W. Betts , F. Cheng , M. G. Francesconi , S. M. Kelly , A. Kornherr , T. J. Prior , J. D. Wadhawan , RSC Adv. 2023, 13, 11884.37077260

[19] a) S. Hou , X. Wang , X. Dong , J. Zheng , S. Li , J. Colloid Interface Sci. 2019, 554, 658. 31351336 10.1016/j.jcis.2019.07.049

[20] a) W. Wang , Y. Zhang , X. Yang , H. Sun , Y. Wu , L. Shao , Engineering 2023, 25, 204;

[21] Y. Zhao , Y. Li , S. Yuan , J. Zhu , S. Houtmeyers , J. Li , R. Dewil , C. Gao , B. Van der Bruggen , J. Mater. Chem. A 2019,7, 6348.

[22] a) Y. Xu , G. Peng , W. Li , Y. Zhu , Z. Mai , N. Mamrol , J. Liao , J. Shen , Y. Zhao , J. Membr. Sci. 2022, 647, 120290;

[23] a) M. Qiu , C. He , J. Hazard. Mater. 2019, 367, 339; 30599406 10.1016/j.jhazmat.2018.12.096

[24] L. Wu , Y. Xu , L. Cai , X. Zang , Z. Li , Appl. Surf. Sci. 314, 2014, 832.

[25] Y. Chen , C. Feng , Q. Zhang , G. Ren , Q. Han , Appl. Surf. Sci. 2019, 467, 526.

[26] N. Sahiner , H. Ozay , O. Ozay , N. Aktas , Appl. Catal. B 2010, 101, 137.

[27] J. Wang , L. Qin , J. Lin , J. Zhu , Y. Zhang , J. Liu , B. Van der Bruggen , Chem. Eng. J. 2017, 323, 56.

[28] a) A. K. Ghosh , B. H. Jeong , X. Huang , E. M. V. Hoek , J. Membr. Sci. 2008, 311, 34;

[29] B. Luo , Y. Yao , J. Liao , Q. Chen , H. Ruan , J. Shen , Adv. Mater. Interfaces 2021, 8, 2100457.

[30] J. Sun , B. Zhang , B. Yu , B. Ma , C. Hu , M. Ulbricht , J. Qu , Environ. Sci. Technol. 2023, 57, 1520. 10.1021/acs.est.2c08707 36630187

[31] a) T. Sun , J. Huang , W. Zhang , X. Zheng , H. Wang , J. Liu , H. Leng , W. Yuan , C. Song , Bioact Mater 2023, 21, 44; 36017072 10.1016/j.bioactmat.2022.07.028 PMC9395756

[32] M. Chen , Y. Cai , G. Li , H. Zhao , T. An , Appl. Catal. B 2022, 307, 121200.

[33] P. S. Goh , A. K. Zulhairun , A. F. Ismail , N. Hilal , Desalination 2019, 468, 114072.

[34] a) A. Elbourne , S. Cheeseman , P. Atkin , N. P. Truong , N. Syed , A. Zavabeti , M. Mohiuddin , D. Esrafilzadeh , D. Cozzolino , C. F. McConville , M. D. Dickey , R. J. Crawford , K. K. Zadeh , J. Chapman , T. Daeneke , V. K. Truong , ACS Nano 2020, 14, 802; 31922722 10.1021/acsnano.9b07861

[35] C. Zhang , Y. Ou , W. Lei , L. Wan , J. Ji , Z. Xu , Angew. Chem., Int. Ed. 2016, 55, 3054. 10.1002/anie.201510724 26822393

[36] Y. Wu , N. Zhang , G. Yuen , C. F. Lannoy , Chem. Eng. J. 2023, 455, 140624.

[37] Y. Li , Y. Cao , D. Jia , J. Mater. Chem. A 2014, 2, 3761.

[38] Y. Chen , R. Sun , W. Yan , M. Wu , Y. Zhou , C. Gao , Sci. Total Environ. 2022, 817, 152897. 35031372 10.1016/j.scitotenv.2021.152897

[39] a) J. Liao , J. Zhu , S. Yang , N. Pan , X. Yu , C. Wang , J. Li , J. Shen , J. Membr. Sci. 2019, 574, 181;

[40] a) I. Wonpil , Biophys. J. 2016, 110, 328;

