# Supplementary material for: Rechargeable Multifunctional Anti‐Bacterial AEMs for Electrodialysis: Improving Anti‐Biological Performance via Synergistic Antibacterial Mechanism
Source: Adv Sci (Weinh). 2023 Sep 11;10(30):2303588. doi: 10.1002/advs.202303588 (PMC10602572; doi:10.1002/advs.202303588)
Supplement: Supplementary file 1 — Supporting Information [file ADVS-10-2303588-s002.pdf]

ADVANCED SCIENCE
Open Access

Supporting Information

for *Adv. Sci.*, DOI 10.1002/advs.202303588

Rechargeable Multifunctional Anti-Bacterial AEMs for Electrodialysis: Improving Anti-Biological Performance via Synergistic Antibacterial Mechanism

Yuyang Yao, Junjie Mu, Yuan Li, Yanjing Ma, Jingwen Xu, Yuna Shi, Junbin Liao, Zhenlu Shen and Jiangnan Shen**

Supporting Information

Rechargeable multifunctional anti-bacterial AEMs for electrodialysis: improving anti-biological performance via synergistic antibacterial mechanism

Yuyang Yao[1], Junjie Mu[1], Yuan Li[2], Yanjing Ma[2], Jingwen Xu[1], Yuna Shi[3], Junbin Liao[1], Zhenlu Shen[1*], Jiangnan Shen[1*]

[1] College of Chemical Engineering, Zhejiang University of Technology, Hangzhou 310014, China;

[2] Information Materials and Intelligent Sensing Laboratory of Anhui Province, Institutes of Physical Science and Information Technology, Anhui University, Hefei 230601, China.

[3] College of Biotechnology and Bioengineering, Zhejiang University of Technology, Hangzhou 310014, China;

[*]Correspondence and requests for materials should be addressed to Prof. Z. Shen (email: zhenlushen@zjut.edu.cn) or to Prof. J. Shen (email: shenjn@zjut.edu.cn).

1.1. ATR-FTIR and XPS

Spectroscopic testing was performed on dried AEM samples using attenuated total reflection Fourier transform infrared spectroscopy (ATR-FTIR) on a Nicolet 6700 spectrometer (US), with a spectral resolution range of 600-4000 cm^{-1}. Surface elemental composition of the AS membrane was determined using X-ray photoelectron spectroscopy (XPS, Thermo Science K-Alpha instrument, USA), which can analyze surface and binding energies within the range of 0-1350 eV. Additionally, all samples were vacuum-dried at 40 ℃ for 24 hours to avoid interference from residual water during the experiment.

1.2. Surface morphology of AEMs

The surface morphology of the prepared ion exchange membranes (IEMs) was observed and investigated by scanning electron microscope (SEM, SU8010, Hitachi, Japan) and Energy Dispersive X-Ray Spectroscopy (EDX, Hitachi HT 7700, Japan). The surface roughness of the membrane was obtained from an atomic force microscope (AFM, Dimension Icon, Bruker, Germany). Prior to testing, the samples were dried under vacuum at 50°C for 48 hours.

1.3. Ion exchange capacity and Swelling ratio

Ion exchange capacity (IEC) is one of the parameters to measure the density of ion exchange groups inside the IEM. The IEC of AEM in Cl^- form was measured by titration. The AEM was soaked in 0.5 M NaCl aqueous solution, to completely replace ions. After 24 h, the IEM was removed and the residual NaCl solution on surface was thoroughly removed with DI water, and then it was exposure to 60 mL of 0.5 M Na_2SO_4 solution for 24 h. As a result, the Cl^- in AEMs was completely released into the solution. The concentration of Cl− was tested via titration by using 0.05 M $AgNO_3$ solution (K_2CrO_4 as indicator). The IECs of AEMs were calculated according to Formula (1):

$$IEC = \frac{V \times c}{W_{dry}} \tag{1}$$

where V is the volume of $AgNO_3$ solution used for titration, in mL; c is the concentration of $AgNO_3$ solution (0.01 M), in mmol/mL; and W_{dry} tests the dry membrane mass of the membrane, in g.

The swelling rate (SR) of the prepared IEMs was determined by measuring the size change before and after full hydration. IEM samples were dried at 50 °C vacuum for 24 hours. The size (area and thickness) of the dried IEM sample was then measured. Soak the dried IEM sample in deionized water (DI water) at room temperature for 24 hours to reach equilibrium, then quickly remove the sample and wipe the deionized water on the surface of the IEM sample with a clean paper towel. And again the size of the hydrated IEM sample is measured quickly and accurately. SR is calculated by Formula (2) :

$$SR = \frac{L_{wet} - L_{dry}}{L_{dry}} \times 100\% \quad (2)$$

where L_{dry} and L_{wet} represent the weight and length of IEM under dry conditions, respectively; W_{wet} and L_{wet} represent the weight and length of IEM under wet conditions, respectively.

1.4. Surface area resistance

AEM samples were exposure to 0.5 M NaCl solution for 12 h before the test, clamped between two compartments, with 0.5 M NaCl solution passing through the middle two compartments and 0.3 M Na_2SO_4 electrode compartment liquid. The voltage on both sides of the AEM is measured at a specified constant current of 0.05 A. The surface area resistance was calculated based on Formula (3) :

$$R = \frac{U - U_0}{I} \times S \quad (3)$$

where U (V) is the trans-membrane voltage; U_0 (V) is the voltage of the blank (V); I (A) is the constant current through the IEMs (A); S is the effective area of IEMs.

1.5. Transport number, Current efficiency and Energy consumption

Transport number was calculated based on the tested potential, which was tested by using the setup containing two compartments filled with the KCl solution with concentration of 0.1 M or 0.2 M. Also, it has been illustrated in our previous work[1]. Before testing, the AEMs was exposure to 0.15 M KCl solution for 0.5 h. Then, as-tested AEMs was clamped by the two compartments. In addition, two Ag/AgCl electrodes were placed on both sides of AEMs to be tested to measure the potential. The ends of the two electrodes were connected to a multimeter (DMM6000, Zhiyuan Electronics Co., Ltd.) to record the potential values at both ends of the AEM. Thus, *t* is calculated as shown in Formula (4) :

$$t = \frac{E_m + E_0}{2E_0} \quad (4)$$

where E_m is the potential at both ends of the AEM, mV; E_0 is the potential difference between 0.1 and 0.2 M KCl standard solution at 25 °C, −16.1 mV.

The electrodes at both ends of the device were externally connected to the power supply, and a constant current of 0.30 A was applied. The effective area of the as-prepared AEM in ED was 19.625 cm^2. The ion conductivities of the NaCl solution in concentrate compartment and dilute compartment were recorded at various time intervals. Finally, the current efficiency (η, %) and energy consumption (E, kWh/kg) of EDs with AEMs were calculated according to Formula (5) and (6):

$$\eta = \frac{zF(c_t - c_0)V_t}{NI\Delta t} \times 100\% \quad (5)$$

$$E = \int_0^t \frac{UI}{c_t V_t M_b} dt \quad (6)$$

Where F is the Faraday constant, (F = 96485 C/mol); c0 and ct are concentration of Na^+ in concentrate compartment at initial and final stage, respectively, (M); V_t is the volume of liquid in concentrate compartment, (L); N is the charge of electrons (N = 1); I is the current intensity, (A); U is voltage across the ED device, (V); M_b is the molecular weight of NaCl, (g/mol).

1.6. Optical density test at Bacterial Eluent

To evaluate the anti-biological reproduction ability of modified AEMs, the optical density (OD) of the bacterial solution after contact with the membrane was tested. All as-tested membranes were cut into square with 1.5 cm sides. In addition, all samples were fully sterilized and operated in clean bench to prevent external bacterial interference. E. coli and S. aureus were used as test bacteria in this experiment and their OD (Synergy LX, BioTek, USA, UV at 600 nm) value was diluted with liquid LB to 0.1. After being well shaken, the supernatant was taken and the OD value was measured. Each sample was immersed in a 12-well plate with 1 mL bacterial solution, and OD values were detected at 12 h and 24 h, respectively.

1.7. Culture plates method

The samples (UV sterilization for 30min) with the size of 1.5 cm × 1.5 cm were placed in a 12-well plate. 100 μL *E. coli* or *S. aureus* was moved to the membrane surface and covered with another membrane. The bacteria were sandwiched by two pieces of films, to completely exposed to the modified functional layer for 1 h, and then fully cleaned with 900 μL PBS1.

1.8. ASTM E2149 methods

The concentration of *E. coli* and *S. aureus* already cultured was controlled at 3×10^5 CFU/mL (PBS1 dilution, quantification by a microplate reader Synergy LX, BioTek, USA, UV at 600 nm). The ratio of membrane to bacterial solution (3×10^5 CFU/mL) is 1 g : 50 mL, it was shaking in a shaker at 37 ^{o}C for 1 h. Then, the mixed solution was diluted 100-times to coat the LB plate and incubate at 37 ^{o}C for 24 h (Note: the control group was selected from the coated plates without incubation and incubation for at 37 ^{o}C for 1 h). Furthermore, each set of experiments was repeated 3 times and the number of colonies in the LB plate was counted and averaged by ImageJ (manual counting). Colony-forming units per millilitre (CFU/mL) were similarly imputed back. Thus, the bacteria reduction and Log_{10} bacteria reduction are calculated as shown in Formula (7) and Formula (8):

$$\text{Reduction}, \% \ (\text{CFU/mL}) = \frac{C-A}{C} \times 100\% \qquad (7)$$

$$\text{Log}_{10}\ \text{bacteria reduction} = \text{Log}_{10}\ (C) - \text{Log}_{10}\ (A) \qquad (8)$$

Where A = CFU per millilitre for the flask containing the treated substrate membrane (Fuji membrane) after the specified contact time, and C = CFU per millilitre for the flask containing the untreated substrate (Fuji-L-CoS-Na or Fuji-L-CoS-Na-Cl membrane) after the specified contact time.

1.9. Crystal violet test

The specific operation consists of the following six steps (**Figure 5**□): In the first stage, 100 μL bacterial solution and membrane (0.3 × 0.4 cm) were placed in 96-well plate, and the OD of diluted bacterial solution was 0.05. After shaking evenly, remove the plate in a 37 °C incubator and culture for X hours (X = 12, 18, 24 and 36). In the second stage, At the end of cultivation, the bacterial solution is completely sucked out by the pipette gun. In the third stage, 0.2 mL methanol was added to each well to immobilize the bacteria and immobilized for 20 min. In addition, the pipette should not touch the membrane material when put the pipette. In the fourth stage, 200 μL 0.1% crystal violet solution was added to each well and stained for 20 min. In the fifth stage, each well was slowly cleaned three times with 0.5 mL PBS, and the crystal violet solution was sucked clean (air dry for two minutes). In the last stage, 200 μL of 95% ethanol solution was added to each well to dissolve and stain crystal violet. After shaking for 5 min, the absorbance of the biofilm was measured at 600nm.

2.Materials

Composition of those different phosphate buffer solution:

PBS: (2.3 g NaH_2PO_4 and 10.9 g Na_2HPO_4 were dissolved in 700 mL DI water); PBS1: (8.0 g NaCl, 0.2 g KCl and 0.24 g Na_2HPO_4 dissolved with 1000 mL DI water);

Commercial AEM (Type-II) were purchased from Fujifilm Corp. The commercial cation exchange membrane (CEM, CMX, ASTOM Co., Japan) and the performance parameters were also tested (see **Table S1**; area resistance: tested in 0.5 M NaCl solution).

Table S1. Some key parameters of commercial IEMs for ED tests.

Membrane	Thickness (μm)	Surface Area Resistance ($\Omega\cdot cm^2$)	pH Stability	IEC
AEM-Type II	175	3.25	2–11	1.52
CMX	160	2.3	4−12	1.67

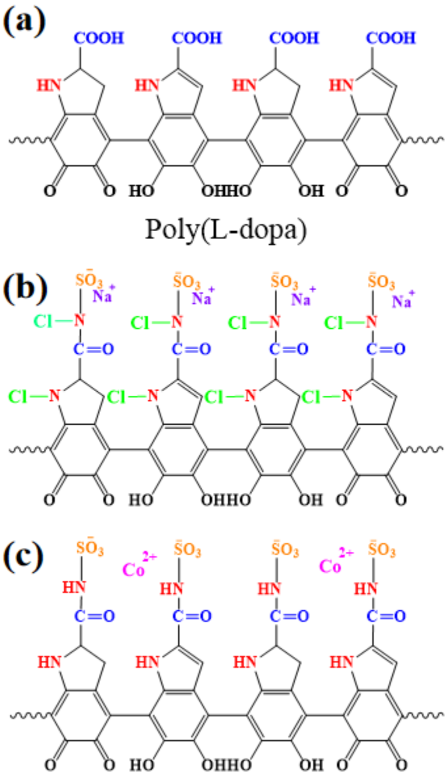

Figure S1. The chemical structure of surface modified layers of Fuji-L, Fuji-L-CoS-Na and Fuji-L-CoS-Na-Cl.

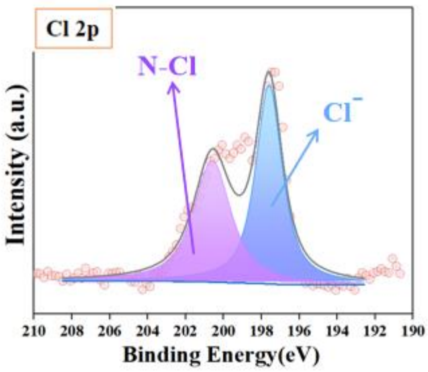

Figure S2. Cl_{2p} high resolution XPS spectrum of Fuji-L-CoS-Na-Cl.

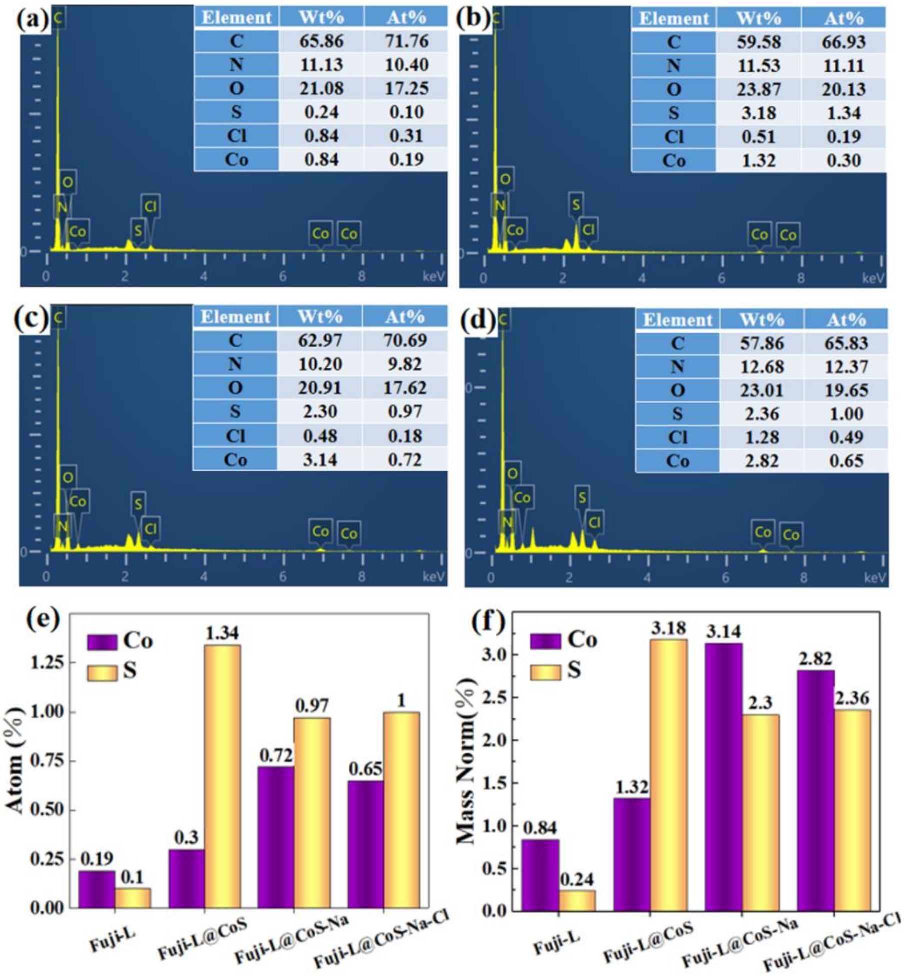

Figure S3. EDX spectrum of the modified membrane: (a) Fuji-L, (b) Fuji-L-CoS, (c) Fuji-L-CoS-Na and (d) Fuji-L-CoS-Na-Cl; Atomic (e) and weight (f) percentage of Co and S elements on the surface of as-prepared AEMs.

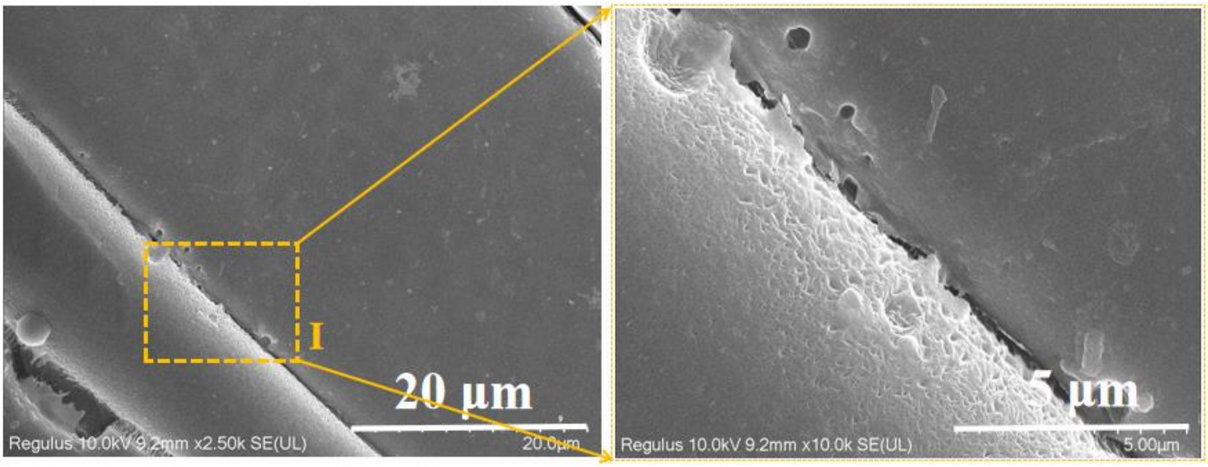

Figure S4. SEM images of Fuji-L-CoS.

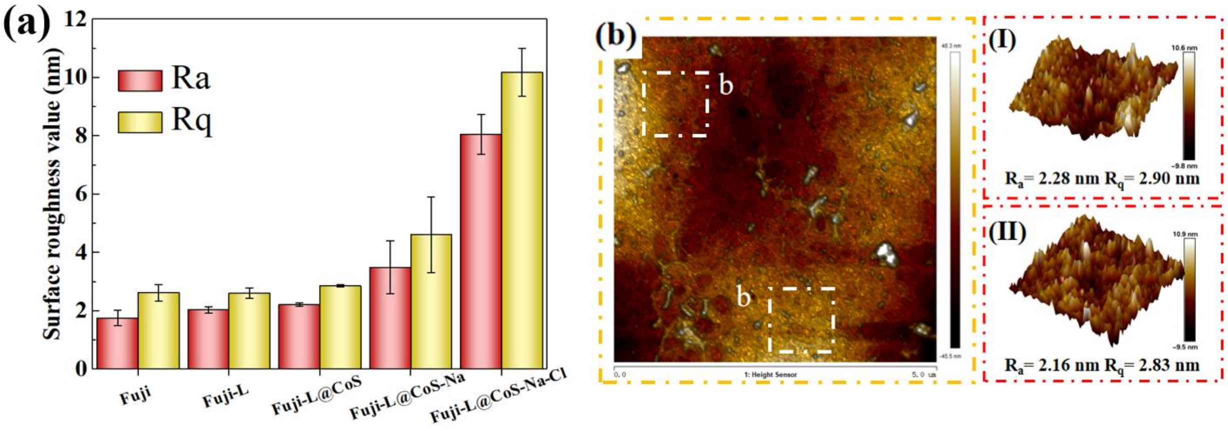

Figure S5. Average surface roughness values (a) of the five membranes, AFM images of as-prepared AEMs surface: (b) Fuji-L-CoS.

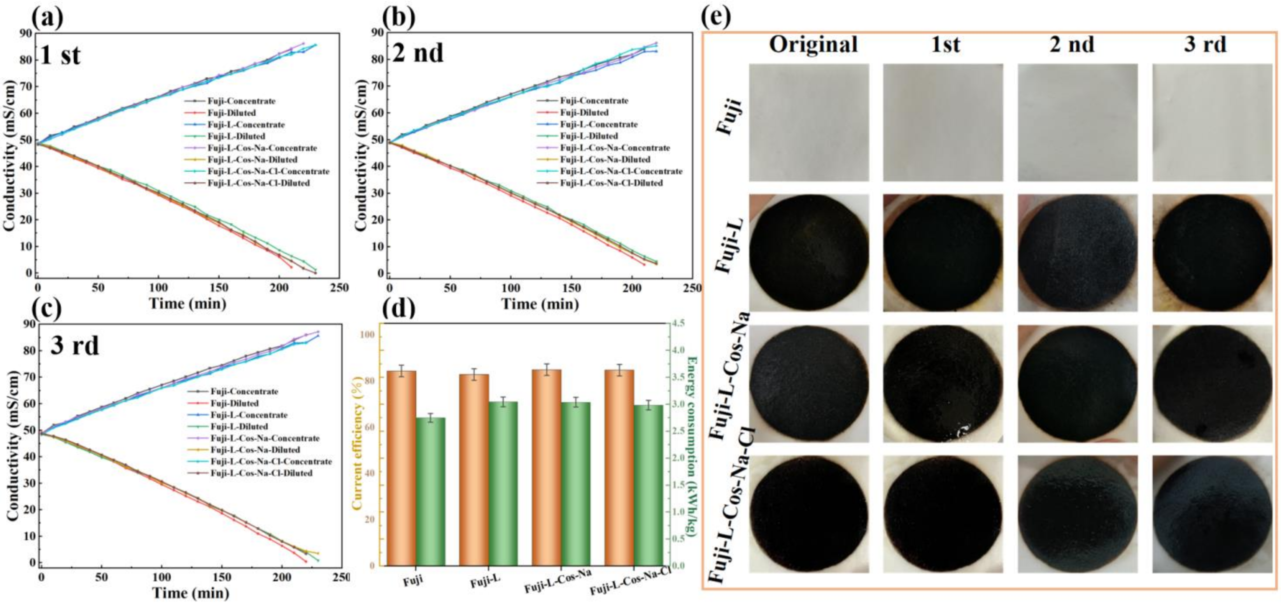

Figure S6. Changes in conductivity during desalination of Nacl solution by Fuji, Fuji-L, Fuji-L-CoS-Na and Fuji-L-CoS-Na-Cl with time: (a) The first time; (b) The second time ; (c) The third time. (d) Mean current efficiency and energy consumption of ED process with Fuji, Fuji-L, Fuji-L-CoS-Na and Fuji-L-CoS-Na-Cl. (e) Photographs of the Fuji, Fuji-L, Fuji-L-CoS-Na and Fuji-L-CoS-Na-Cl after three times of ED desalination. Current density: 15.29 mA·cm^{-2}; Temperature: 22 ^{o}C ± 3 ^{o}C.

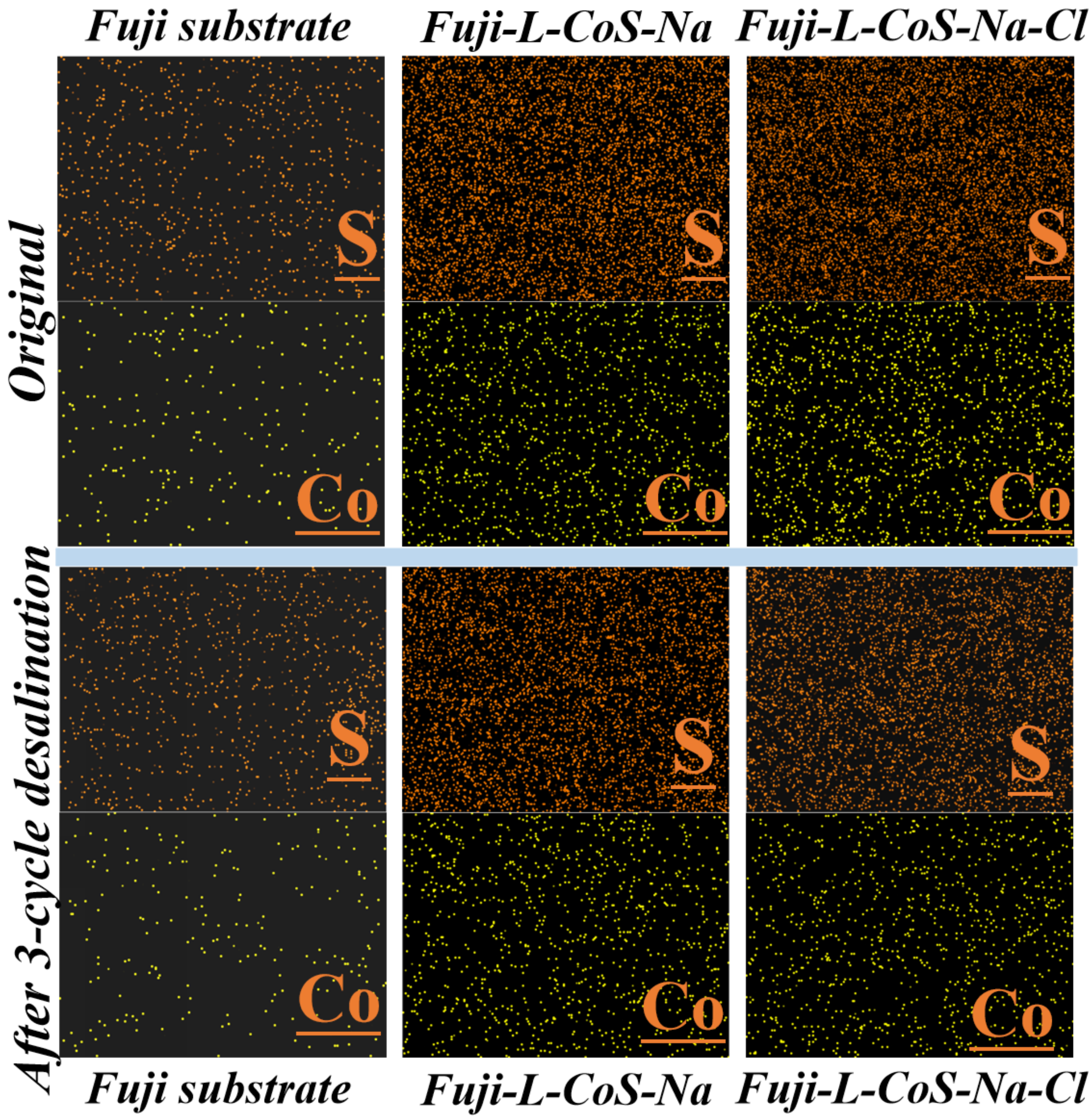

Figure S7. The energy-dispersive X-ray spectroscopy elemental maps of S and Co on the Fuji, Fuji-L-CoS-Na and Fuji-L-CoS-Na-Cl membrane surface (Original and after 3-cycle desalination membrane).

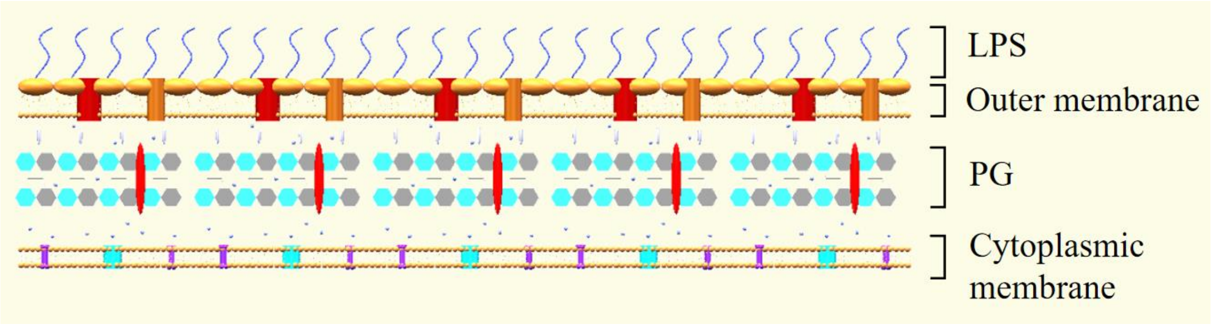

Figure S8. The composition of the Gram-negative cell envelope.

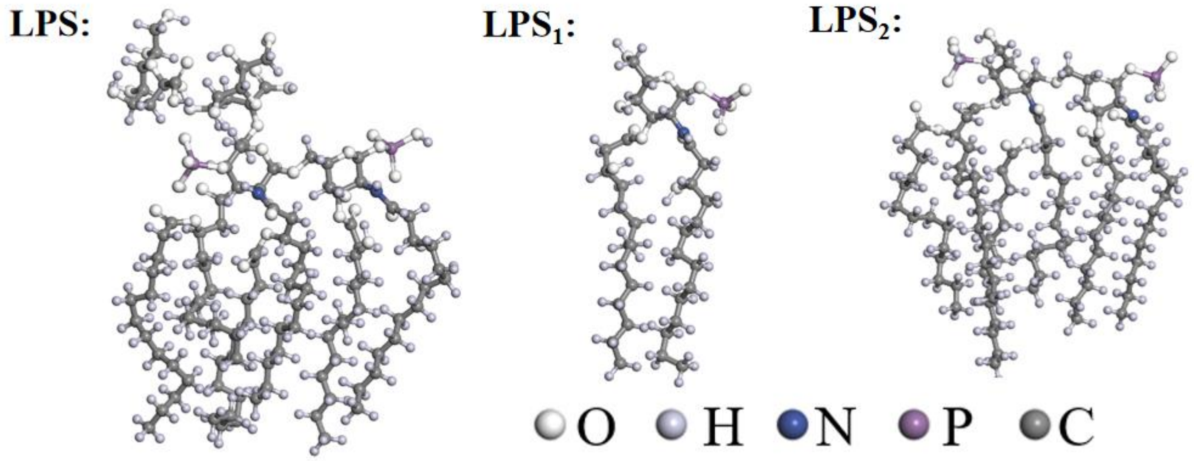

Figure S9. The simplified structures of LPS, LPS_1 and LPS_2.

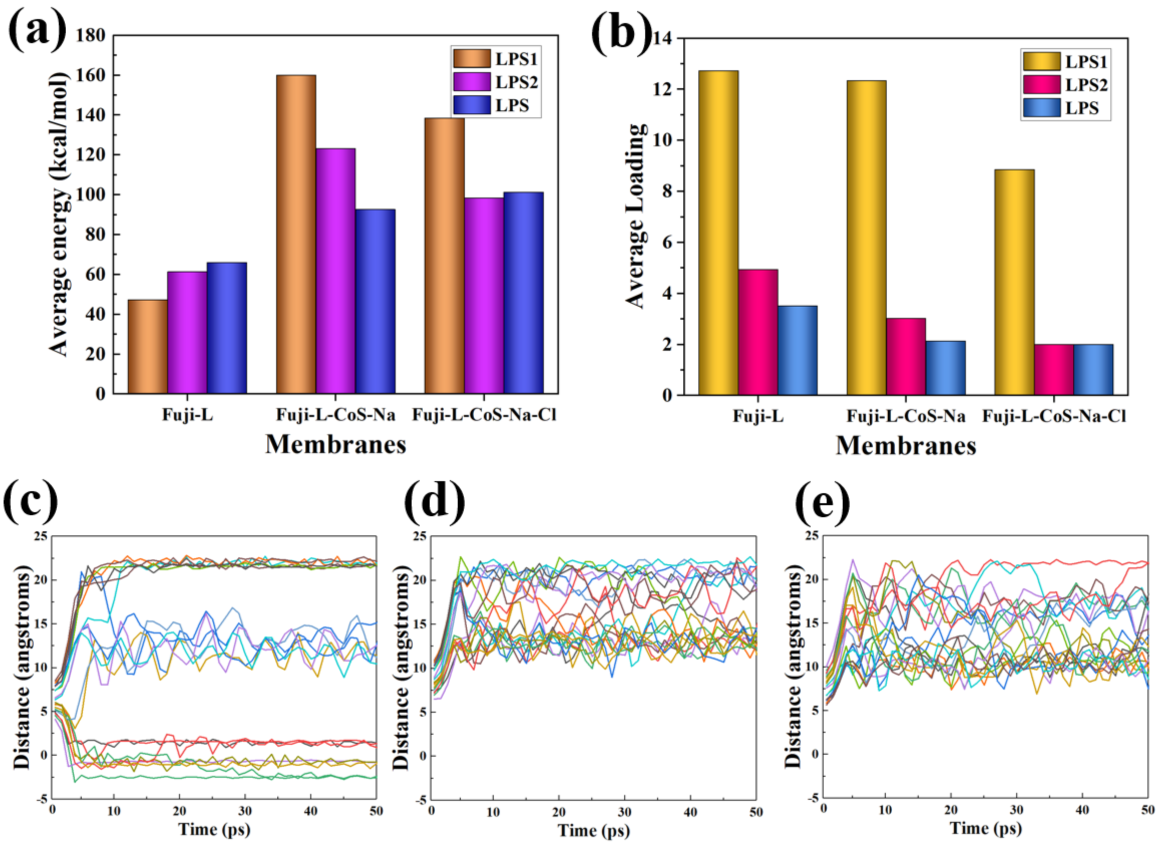

Figure S10. (a) The average adsorption energy and (b) average loading of these three modified layers; The movement trajectories of 20 PA_1 on the (c) Fuji-L, (d) Fuji-L-CoS-Na and (e) Fuji-L-CoS-Na-Cl within 50 ps.

Table S2. ASTM E2149 method (*E. coli*) for as-prepared membranes.

	**Control 0s**	**Control 1h**	**Fuji 1h**	**Fuji-L-CoS-Na 1h**	**Fuji-L-CoS-Na-Cl 1h**
A group	663	373	971	6	3
B group	651	1032	297	5	1
C group	729	1121	1396	1	2
Average value	681	842	888	4	2
Colony-forming units per millilitre (CFU/mL)	681000	842000	888000	4000	2000
Percent bacterial reduction(%)	/	/	/	99.55	99.77
Mean Log_{10} density of bacteria (Log_{10} CFU/mL)	5.83	5.93	5.95	3.60	3.30
Mean Log_{10} bacterial reduction (Log_{10} CFU/mL)	/	/	/	2.35	2.65

Table S3. ASTM E2149 method (*S. aureus*) for as-prepared membranes.

	**Control 0s**	**Control 1h**	**Fuji 1h**	**Fuji-L-CoS-Na 1h**	**Fuji-L-CoS-Na-Cl 1h**
A group	62	/	48	0	0
B group	58	/	39	0	0
C group	52	/	51	0	0
Average value	57.33	/	46	0	0
Colony-forming units per millilitre (CFU/mL)	57333.33	/	46000	0	0
Percent bacterial reduction(%)	/	/	/	100.00	100.00
Mean Log_{10} density of bacteria (Log_{10} CFU/mL)	4.76	/	4.66	/	/
Mean Log_{10} bacterial reduction (Log_{10} CFU/mL)	/	/	/	4.66	4.66

[1] J. Liao, J. Xu, H. Ruan, J. Mu, X. Jie, W. Li, Y. Xu, J. Shen. *Desalination*, **2023**, 546, 116202.
